# Observation-Based Estimates of Global Glacier Mass Change and Its Contribution to Sea-Level Change

**DOI:** 10.1007/s10712-016-9394-y

**Published:** 2016-11-11

**Authors:** B. Marzeion, N. Champollion, W. Haeberli, K. Langley, P. Leclercq, F. Paul

**Affiliations:** 1 Institute of Geography, University of Bremen, Postfach 330 440, 28334 Bremen, Germany; 2 International Space Science Institute, Hallerstrasse 6, 3012 Bern, Switzerland; 3 Department of Geography, University of Zurich, Winterthurerstrasse 190, 8057 Zurich, Switzerland; 4 Asiaq Greenland Survey, Qatserisut 8, 3900 Nuuk, Greenland; 5 Department of Geosciences, University of Oslo, P.O. Box 1047, Blindern, 0316 Oslo, Norway

**Keywords:** Glacier mass change, In situ and satellite observations, Glacier modeling, Mean sea level

## Abstract

Glaciers have strongly contributed to sea-level rise during the past century and will continue to be an important part of the sea-level budget during the twenty-first century. Here, we review the progress in estimating global glacier mass change from in situ measurements of mass and length changes, remote sensing methods, and mass balance modeling driven by climate observations. For the period before the onset of satellite observations, different strategies to overcome the uncertainty associated with monitoring only a small sample of the world’s glaciers have been developed. These methods now yield estimates generally reconcilable with each other within their respective uncertainty margins. Whereas this is also the case for the recent decades, the greatly increased number of estimates obtained from remote sensing reveals that gravimetry-based methods typically arrive at lower mass loss estimates than the other methods. We suggest that strategies for better interconnecting the different methods are needed to ensure progress and to increase the temporal and spatial detail of reliable glacier mass change estimates.

## Introduction

Glaciers are distinctive features of many high-altitude and high-latitude landscapes around the world. Their geometric response to changes in atmospheric conditions is slow enough to filter out high-frequency weather and climate variability, but fast enough to provide humans with visible impressions of systematic changes of the environment, without the need for technical or statistical tools. Because of these properties, glaciers have become one of the key indicators of climate change (e.g., WGMS 2008; Marzeion et al. 2014a). Perhaps more importantly, glaciers are closely linked to the Earth system not only by being shaped by atmospheric conditions and their topographic setting, but by changing the seasonality of water runoff in many large river systems (e.g., Immerzeel et al. 2010; Kaser et al. 2010; Huss 2011), by being central to many geomorphologic processes (e.g., Egholm et al. 2009; Korup et al. 2010; Thomson et al. 2010; Koppes et al. 2015; Haeberli et al. 2016) and by affecting sea level through changes of the terrestrially stored water mass (see e.g., Radić and Hock 2010; Huss and Farinotti 2012; Grinsted 2013 for current estimates of mass stored in glaciers). Of interest here are changes to the mass of water stored in glaciers on the global scale, thus affecting the global mean sea level, over a time scale of several years (i.e., excluding seasonal mass changes, Jansson et al. 2003).^1^ Methods to derive glacier mass changes from in situ and remote sensing observations are presented and compared in order to obtain a globally coherent picture from spatially incomplete and temporarily inconsistent datasets.

The importance of glacier mass change for sea-level change is a function of the time scales and spatial scales considered: on decadal and shorter time scales, and on small spatial scales, sea-level variability is dominated by ocean dynamics and wind stress (e.g., Meyssignac and Cazenave 2012; Richter et al. 2012). On longer time scales and small spatial scales, glaciers may contribute to distinct patterns in relative sea-level change through mass redistribution and isostatic effects (e.g., Tamisiea et al. 2003; Larsen et al. 2005; Melini et al. 2015). Finally, long-term, systematic changes to glacier mass will impact their relative importance for the global sea level. That is, in globally cool periods with large glaciers, small temperature changes can lead to relatively large sea-level change from glaciers, while in warm periods with small glaciers, even large temperature change has a limited impact on glacier-related sea-level change (Marzeion et al. 2014b). Because of this nonlinearity, glaciers will likely play a lesser role for sea-level change in the twenty-first century than they did within the twentieth century, but still be a main contributor along with ice sheet mass loss and thermal expansion of sea water (Meier et al. 2007; Church et al. 2013; Allison et al. 2014).

In order to be able to assess our understanding of sea-level change, knowledge of glacier mass change is therefore critical. In recognition of this relevance, glaciers were identified as an Essential Climate Variable (ECV) in the terrestrial component of the Global Climate Observing System (GCOS) in support of the United Nations Framework Convention on Climate Change (UNFCCC). Observations of glaciers are organized within the Global Terrestrial Network for Glaciers (GTN-G), with the corresponding work being coordinated between the World Glacier Monitoring Service (WGMS; mainly responsible for in situ observations), the Global Land Ice Measurement from Space initiative (GLIMS, satellite observations, Raup et al. 2007) and the National Snow and Ice Data Center at Boulder/Colorado (NSIDC, data management and photo collection).

While the observational network is rapidly growing, data on glaciers are characterized by a severe sub-sampling problem on the global scale: whereas there are individual time series of direct observations of glacier mass change reaching back to the ninetieth century (see Sect. 2.1), they necessarily represent a very small sample of the roughly 200,000 glaciers worldwide (Pfeffer et al. 2014). Glacier length change observations (presented in Sect. 2.2) are often available for even longer time periods and valuable for contextualizing the shorter, direct observations of mass change, but the link between glacier length and mass changes is complicated through ice dynamics and is strongly non-linear, and thus needs careful calibration. Remote sensing techniques of glacier area and volume change (see Sects. 3.1, 3.2) are comprehensive and spatially detailed, but available on the global scale for only relatively short time. Moreover, conclusions on associated ice mass change are not simple to draw, as both glacier volume and area change first need to be related to ice mass (by estimating ice density or ice thickness, respectively) or have to be derived by other means (e.g., Glasser et al. 2011). Direct remote sensing of glacier mass change (see Sect. 3.3) is a relatively recent and promising observation method. As of now, time series are short and the spatial resolution is coarse (a few hundred km), complicating the separation of the glacier mass change signal from other mass changes. Finally, it is possible to use observations of the state of the atmosphere in order to force a glacier model to obtain estimates of glacier mass change (see Sect. 4). Modeling of glaciers allows for comprehensive and long-term assessments. However, the quality of the model results critically depends on model reliability and the quality of atmospheric observations, which generally speaking are of questionable quality in glacierized regions because of their remoteness and terrain complexity. In the discussion (Sect. 5), we critically review the efforts to synthesize these different approaches and indicate recent achievements and the most pressing gaps.

## Long-Term Glacier Monitoring

### Glacier Mass Change

Data on glacier mass balance and length fluctuations document changes in time, often at high temporal resolution (annually), while glacier inventories, repeated at time intervals of a few decades, enable an assessment of how representative this information is in space. Glacier mass balance is commonly considered to be an undelayed response to atmospheric forcing, whereas glacier length change represents a filtered, enhanced and cumulative but also delayed response to a longer-term forcing, taking place at the temporal scale of climate change (i.e., decades to millennia). This delayed response of geometric adjustment to changes in climatic conditions and glacier mass balance (“response time”; decades for steep/mid-size glaciers to centuries for the flat/largest glaciers, see, e.g., Jóhannesson et al. 1989; Haeberli and Hoelzle 1995; Bahr et al. 1998; Harrison et al. 2001) induces a feedback on glacier mass balance (via mass balance-altitude effects). For the current measurement network, emphasis is put on calibrated long-term mass balances of entire glaciers investigated with direct field measurements (stakes and snow pits), enabling a detailed process understanding (accumulation-ablation, comparison with meteorological information) at high resolution in time (seasons, years) and with repeated precision mapping to determine volume/mass changes for the entire glacier.

The study by Zemp et al. (2015) provides the most recent summary of in situ observations collected by the World Glacier Monitoring Service (WGMS). They conclude that rates of early twenty-first century mass loss are historically unprecedented for the worldwide sample of observed glaciers and mainly driven by ablation processes. For the sample of long and continuous mass balance time series, rates of mass loss increased from about 0.2 m water equivalent (w.e.) per year in the decade 1980–1990 to about 0.4 m w.e. per year in the decade 1990–2000 to about 0.8 m w.e. per year in the first decade of the twenty-first century. Besides this strong evidence for accelerated climate change, the worldwide uniformity of the signal is striking: with very few exceptions (e.g., Karakoram, Pamir, see Cogley 2009; Kääb et al. 2012; Gardner et al. 2013), glaciers are shrinking rapidly and at a global scale. It is this clarity which makes glaciers “unique demonstration objects of climate change” (WGMS 2008) and key indicators in global climate system monitoring. Locally and regionally, differential change patterns take place: in case of continued atmospheric warming, glaciers with short response times can adjust quickly and remain relatively close to equilibrium conditions, while glaciers with long response times increasingly depart from equilibrium conditions (Haeberli and Hoelzle 1995). As a result, glaciers with long response times are now far too large as compared to equilibrium with twenty-first century climatic conditions and show massive down-wasting in many regions of the world (Larsen et al. 2007; Paul and Haeberli 2008; Gardelle et al. 2013). Detailed repeat inventories in well-documented regions indeed show a tendency for larger glaciers to have higher mass losses than smaller glaciers, but statistics relating rates of mass loss to geometric and climatic parameters generally exhibit large scatter and complex spatial patterns (Fischer et al. 2015).

The question of representative mass balance time series can be treated on the basis of these results, where one has to distinguish between two types of representativeness: concerning representation of the climate signal at high time resolution, well-calibrated mass balances from relatively small and comparatively steep glaciers with short response times are most significant. However, sea level is predominantly influenced by the largest and comparatively flat glaciers on Earth with long response times (Meier et al. 2007), thus requiring a different set of representative glaciers. Results from one set cannot be directly translated to the other (e.g., Le Bris and Paul 2015), because the strong or even extreme dis-equilibrium of large glaciers exerts a strong feedback on rates of mass loss via the mass balance-altitude feedback (Raymond et al. 2005). For total glacier mass change, Cogley (2009 and updates thereof) developed a scheme for weighting the spatially inhomogeneous observations and combining geodetic and direct mass balance measurements.

The marked dis-equilibrium of (especially large/flat) glaciers means that there is a strong commitment for future mass loss (Mernild et al. 2013; Marzeion et al. 2014b). In the European Alps, for instance, about half of the still existing glacier area will have to disappear in order for glaciers to adjust to climatic conditions during the first decade of the twenty-first century (Carturan et al. 2013a). Also in other similar cases, i.e. in many mid-latitude mountain ranges, the option of “saving the glaciers” hardly exists anymore because most of the ice will have disappeared within the coming decades already, before measures of global climate policy could show significant effects. Mernild et al. (2013) estimate the committed, but not yet realized, mass loss of glaciers globally at 163 ± 69 mm sea-level equivalent (SLE) based on the climate of first decade of the twenty-first century, Marzeion et al. (2014b) estimate this number at 66 ± 2 mm SLE, but for the climate of the earlier (and substantially cooler) period of 1961 to 1990.

As a consequence of ongoing rapid glacier vanishing, many of the mass balance glaciers in the international glacier monitoring network are likely to disintegrate or completely disappear within the next few decades (e.g., Carturan et al. 2013b). New mass balance programs must therefore be established on still larger/higher/thicker glaciers to save continuity. The homogenization of long-term series can then be carried out on the basis of repeat glacier inventories and inter-comparison of past melt rates in order to determine the effect of the changed set of observed glaciers. The same technique can also be used to assess quantitative information on mass changes through time of large glacier samples and entire mountain regions (Paul and Haeberli 2008). New technologies like numerical modeling of detailed glacier-bed topographies (e.g., Clarke et al. 2013; Huss and Farinotti 2012; Linsbauer et al. 2012) that can be used to bridge spatio-temporal gaps in the observational network and low-cost/high-precision measurements with LIDAR (Bhardwaj et al. 2016a) or drones (Bhardwaj et al. 2016b) are now refining observational techniques as well as the frequency and quality of obtained results.

### Glacier Length Change

As direct observations of glacier volume and glacier mass change did not have global coverage prior to before the late twentieth century, other observations of glacier changes are needed to determine the glacier contribution to sea-level rise prior to the second half of the twentieth century. Glacier length fluctuations seem to be the most suited type of measurements to get a representative picture of long-term global glacier changes, as direct measurements of length changes (also called front variations) have a long history. When worldwide glacier monitoring began in 1894 with the founding of the International Glacier Commission, collection and publication of frontal fluctuations was the first focus (Zemp et al. 2014). In addition, frontal positions can be reconstructed from geomorphologic landforms (e.g., moraines), biological evidence such as overridden trees and the lifetime of lichens (e.g., Rabatel et al. 2005; Bushueva and Solomina 2012) and historical evidence such as paintings, photos, and early maps (e.g., Zumbühl 1980; Nussbaumer and Zumbühl 2012). Combining all this information has resulted in a comprehensive overview of global glacier fluctuations, also from periods before monitoring programs started.

In all glacierized regions on Earth measurements of glacier length fluctuations are available through in situ measurements, often in connection with long-term monitoring programs or through remote sensing. The first direct measurements originate from the nineteenth century, and the number of measurements strongly increased in the beginning of the twentieth century. Since 2011, WGMS has also collected reconstructed frontal variations (Zemp et al. 2011), so that at present the database contains approx. 40,000 observations of about 2000 glaciers and covers the period from the sixteenth century until the present.

A qualitative analysis of the data included in the WGMS database shows a dominant retreat of glaciers over the last 100–150 years with some intermittent advances (Zemp et al. 2015). The advances are small (a few 100 m) compared to the overall retreat (up to a few km) and asynchronous in the different glacier regions—except for the 1970s, when about a third of the glaciers included in the dataset at that time advanced.

Leclercq et al. (2014) present a worldwide dataset of long-term glacier length changes for 471 glaciers that includes a large compilation of reconstructed historical changes from a large variety of sources which, at the time, were not included in the WGMS (after submission of the data to the WGMS, they are also included in the WGMS dataset). Leclercq et al. (2014) include only long-term, cumulative glacier length records that start prior to 1950 and cover four decades at least. Like Zemp et al. (2015), Leclercq et al. (2014) found a general trend of glacier retreat since the middle of the nineteenth century, despite some intermittent periods of advance for several glaciers. Based on the global dataset of 471 glaciers, the mean twentieth century glacier length reduction is 1.5 km. There is a large variation in the retreat of individual glaciers ranging from 30 m to 23 km over the twentieth century, and a difference in regional averages of length change of the glaciers in the dataset. For example, the mean retreat of Alaskan glaciers in the dataset is 5.18 ± 0.33 km, while the average twentieth century retreat in Scandinavia is 1.11 ± 0.02 km, and in High Mountain Asia is 1.02 ± 0.05 km. The global average rate of retreat in the first half of the twentieth century, 12.5 m year^−1^ in the period 1921–1960, is larger than the retreat in the second half of the twentieth century (7.4 m year^−1^ in the period 1961–2000), which may reflect the shortening of glaciers themselves, as length change can be expected to scale with glacier length (Jóhannesson et al. 1989). On average, calving glaciers retreated much more than land-terminating glaciers, with a twentieth century average retreat of 4.9 and 1.1 km, respectively. Nevertheless, the average relative retreat of calving glaciers is almost identical to the global mean relative retreat of land-terminating glaciers, again confirming the relation between glacier length and length change.

**Fig. 1:**
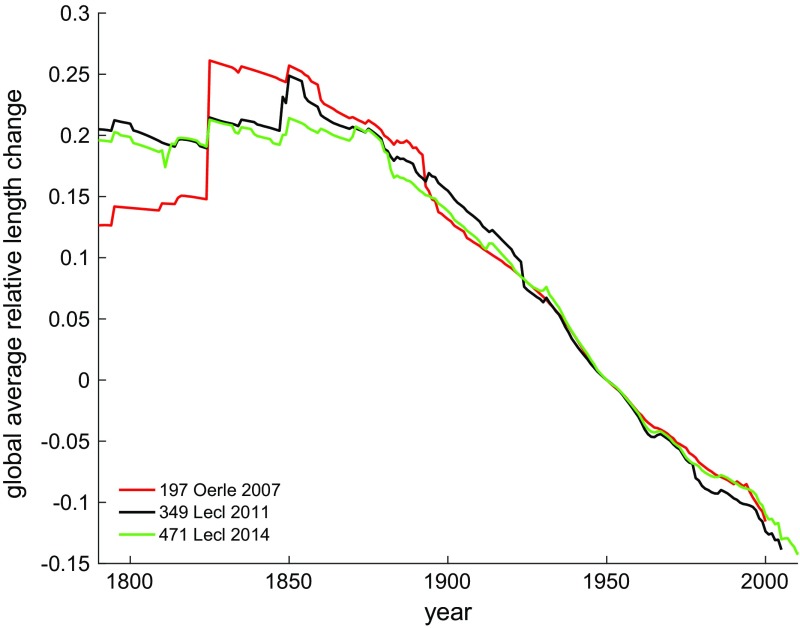
Global average relative length change (i.e., length change normalized to the glacier length in 1950), based on the glaciers included in three versions of the dataset of worldwide glacier length fluctuations as used in Oerlemans et al. (2007, 197 records, *red*), Leclercq et al. (2011, 349 records, *black*) and Leclercq et al. (2014, 471 records, *green*)

The global average of relative glacier length change is remarkably consistent despite the large increase in the number of glacier length records and the corresponding improvement of the global coverage of the glacierized regions. Of the 197 glaciers included in Oerlemans et al. (2007), more than half are located in central Europe and Scandinavia and only 12 in the Arctic. The representativeness of the world’s glaciers has substantially improved in later versions of the dataset by addition of, among others, more than 100 records in the Arctic and around 30 in both the Southern Andes and Central Asia. Despite the large differences in the characteristics of the three datasets, the global average relative glacier length change is fairly similar (Fig. 1), indicating a worldwide coherent glacier change.

Like glacier volume, glacier terminus fluctuations lag and filter variations in the climatic forcing due to the response time of the dynamical glacier system. The length response time is generally a bit larger than the volume response time (Oerlemans 2001), so that the observed length changes cannot directly be translated to volume changes.

Lüthi et al. (2010) use a dynamic equivalent simple model to describe glacier evolution forced by varying equilibrium line altitudes. After calibration of the model parameters, based on the match of modeled and observed length changes, they are able to reproduce time series of observed volume changes for 13 glaciers in Switzerland. In order to produce absolute volumes, the model results have to be scaled with the observed volume change over one time interval for each of the glaciers. However, if applied to a small sample of glaciers, these absolute volume estimates may be relatively uncertain. Oerlemans et al. (2007) proposed a different approach to derive volume change from length observations on a global scale. They combined observed cumulative length changes of 197 glaciers to reconstruct a global length change signal. They used the relative length change, rather than the absolute cumulative length change, in their measure for global length change, as the relative length change gives a globally very coherent signal (see Fig. 1). This global relative length change was scaled to global relative volume change, and the relative volume change is translated into global glacier mass change by calibration against the global glacier mass loss over the period 1961–2003, based on a compilation of glacier mass balance data (Dyurgerov and Meier 2005). Oerlemans et al. (2007) found a glacier contribution to sea-level change of 55 ± 10 mm SLE for the period 1850–2000 (0.37 mm SLE year^−1^), and 45 ± 70 mm SLE for 1900–2000 (0.45 mm SLE year^−1^).

Leclercq et al. (2011) estimated a significantly higher glacier contribution to sea-level rise. Their estimate is based on the same method as used by Oerlemans et al. (2007), but they used extended datasets of global glacier length changes and mass balance observations that provided better global coverage. The mass balance dataset also included available geodetic mass balances (Cogley 2009), and the number of length change records is increased from 197 to 349. The estimated glacier contribution to sea-level change resulted in 91 ± 10 mm SLE for the period 1850–2005 (0.59 mm SLE year^−1^), and 67 ± 16 mm SLE for 1900–2005 (0.64 mm SLE year^−1^). A more recent update, based on even more geodetic and glaciological mass balance observations and glacier length records, results in an even higher estimate of glacier contribution to sea-level rise of 80 ± 21 mm SLE for 1900–2005 (0.76 mm SLE year^−1^), excluding the Antarctic peripheral glaciers (Marzeion et al. 2015). Uncertainty analysis shows that the results are most sensitive to the calibration of the scaled global length signal to the global mass balance observations compilation (Leclercq et al. 2011). The large increase in reconstructed sea-level contribution of glaciers as presented in Oerlemans et al. (2007), Leclercq et al. (2011) and Marzeion et al. (2015) can be ascribed mainly to the increase in the glacier mass loss in the consecutive versions of the global mass balance observations (Dyurgerov and Meier 2005; Cogley 2009 and updates thereof, WGMS 2015).

The development of automated flow-line calculations based on glacier inventory data and digital elevation models (e.g., Kienholz et al. 2014; Machguth and Huss 2014) now enables large numbers of length change data to be collected all over the world, based on satellite information. However, the interpretation of such data is becoming increasingly more difficult as more and more glaciers are turning from a dynamically active retreat to down-wasting, and even collapse, with ill-defined glacier margins. In this regard, glaciers with dynamic instabilities and surge-type glaciers with excessive length changes (km scale) over short periods of time (years) need to be excluded to the greatest extent possible using available databases (Sevestre and Benn 2015, see also Roe 2011).

## Satellite Remote Sensing of Glaciers

The use of space-borne observations for global glacier monitoring has increased dramatically over the past two decades. By all means, satellites provide information that is complementary to field observations, in particular when it comes to complete spatial coverage and remote regions. Moreover, they also provide observations for time periods that are not covered by field observations (Barandun et al. 2015) or products that are difficult to obtain in the field or from airborne remote sensing. For the time period they cover, their particular strength is in global scale observations with consistent and reproducible methods. Key products derived from satellite data are glacier outlines and inventories (in combination with a digital elevation model, DEM, e.g., Andreassen et al. 2012), consistent DEMs of the surface topography with global coverage (e.g., the SRTM DEM or the ASTER GDEM), elevation changes over entire glaciers from differencing DEMs from two epochs or at points from repeat altimetry (e.g., Nuth et al. 2010), surface flow velocities for determination of mass fluxes (e.g., Melkonian et al. 2013, 2016), glacier mass changes from space-borne gravimetry observations (using the GRACE satellites, e.g., Jacob et al. 2012) and glacier facies mapping (ice, firn, snow) that is used as a proxy for mass balance (e.g., Rabatel et al. 2008) and an important input dataset for hydrologic models or for calibration and/or validation of distributed mass balance models (e.g., Immerzeel et al. 2009; Paul et al. 2009).

Apart from elevation changes and flow velocities that are already determined from datasets acquired at two different points in time, area and length changes can also be derived from multi-temporal datasets. Whereas change rates are of interest for a comparison across regions, it can be instructive to look at the developments of trends through time, i.e. acceleration of rates. This has mostly been done with time series of area changes (e.g., Paul et al. 2004b; Klein and Kincaid 2006; Narama et al. 2010; Tennant et al. 2012; Falaschi et al. 2013) but also with changes in velocity (Heid and Kääb 2012; Quincey et al. 2015) or elevation (Holzer et al. 2015). A trend in any of these parameters is most useful for the determination of climate change impacts (e.g., an increase of mass loss or area shrinkage rates through time). However, all of these datasets have to be determined in regard to the area covered by glaciers. Unfortunately, generating precise glacier outlines still requires large amounts of manual work by trained experts, e.g., to delineate their debris-covered parts (see 3.1.2). In consequence, glacier outlines used for calculations are often temporally misaligned with other datasets. A baseline dataset with complete global coverage has only recently been established in the form of the Randolph Glacier Inventory (RGI, Pfeffer et al. 2014),

Since it became available, the RGI was widely applied by the glaciological and hydrological community for a range of applications, including an estimate of the global glacier mass budget as derived from a combination of different methods (Gardner et al. 2013). A key asset of the inventory is the possibility to upscale information that is spatially incomplete (e.g., determined only at points) or heterogeneous (e.g., data voids in DEMs), using relations that can be derived from the individual measurements (e.g., the elevation dependence of mass loss or size class specific area change rates). With careful handling of the different time periods covered by each dataset, it is possible to derive a global picture of glacier mass changes based on field data of selected glaciers (providing the longest time series), geodetic changes from DEM differencing (providing decadal changes for entire mountain ranges), repeat altimetry (point information at sub-annual timescales) and direct mass changes from spaceborne gravimetry (for large regions that are homogenously glacierized). Table 1 gives an overview of some common methods used to derive elevation, volume or mass changes for glaciers. Airborne datasets are not considered in this table, but provide an important means to cover (temporal) data gaps (e.g., Operation IceBridge, Studinger et al. 2010) or calibration data for mass balance time series using the geodetic method and satellite-derived products (e.g., photogrammetric DEMs, Huss et al. 2015; Andreassen et al. 2016).

**Table 1:** Methods that can be used to determine elevation, volume and mass changes of glaciers from satellite data

Survey	Sensor	Change measured	Coverage	Time period	Temporal resolution	Issues	Accuracy
Field data	Stakes and pits, GPR	Elevation, mass	Selected glaciers globally	Since 1947	Annual	Point data, small sample, extrapolation	0.2 m
Repeat altimetry	ICESat, Cryosat2	Elevation	Globally but only along stripes	Since 2003 Since 2012	Sub-annual	Point data, extrapolation	0.5 m
DEM differencing	SRTM, GDEM SPOT/national High-resolution	Volume	Globally, w/o poles Mountain regions Selected glaciers	Around 2000 After 2000, 1960s After 2010	Decadal	Data voids, artifacts	1–2 m Better 1 m Better 0.5 m
Gravimetry	GRACE	Mass	Regional means and globally	Since 2002	Monthly	Coarse resolution	Variable

Field measurements added as reference

Owing to the complementary nature of satellite-derived observations, accuracy assessment and validation by field measurements is difficult. It is generally performed by comparing datasets across sources (field, air, space). For example, DEM quality is determined (and adjusted) using laser altimetry data from ICESat, cumulative mass balances measured in the field are calibrated with geodetic balances derived from DEM differencing, snow lines derived from satellite data are validated by field observations, and mass changes from spaceborne gravimetry are spatially constrained using glacier extents. Hence, all source data and methods have to play multiple roles and are equally valuable. Below, we provide a short overview on how glacier outlines, elevation/volume and mass changes are derived from satellite sensors and present the latest results from global scale assessments.

### Glacier Mapping and Area Changes

#### The Importance of Precise Glacier Extents for the Determination of Glacier Mass Changes

**Fig. 2:**
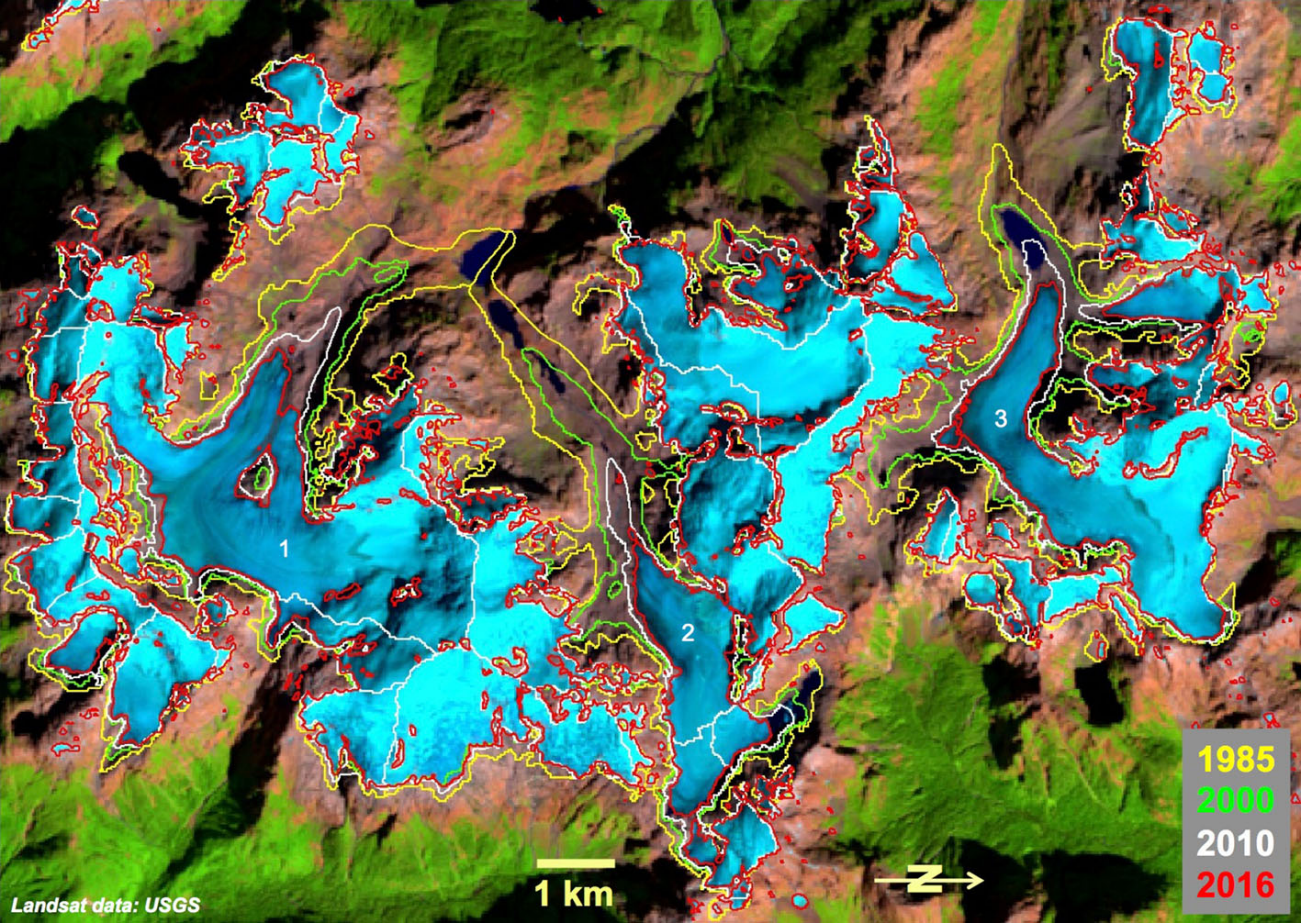
Dramatic glacier shrinkage in northern Patagonia around the volcano Monte Inexplorado from 1985 to 2016. When glaciers retreat kilometers per decade, outlines need to be obtained for the time of DEM acquisition. Good temporal match of datasets (i.e., outlines and DEMs) is essential for correct change deduction. The three largest glaciers flowing to the west have lost 1/3 of their area from 1985 to 2010

As mentioned above, precise glacier extents are key to determining glacier mass changes and thus their contribution to sea-level change. Apart from technical and methodological challenges related to the mapping itself, a key problem of a precise delineation lies in the very nature of glaciers as climatic indicators: their rapid geometric changes in response to climate change and/or dynamic instabilities. This inevitably results in a temporal mismatch of glacier outlines and the datasets that use them to spatially constrain the analysis. Henceforth, all calculations using glacier outlines from a different point in time have an inherent uncertainty that can be smaller (e.g., in regions where glaciers show limited changes over decades) or larger (e.g., for rapidly changing calving or surging glaciers). In Fig. 2, an example is shown with some rapidly shrinking valley glaciers in northern Patagonia that require a very good temporal match of the outlines and the DEM to precisely derive volume loss.

For several applications, it is sufficient to just know where glaciers are located, i.e. a simple (binary) yes/no mask of glacier coverage. Such a glacier mask can be derived rather quickly from satellite imagery (for clean ice) as it is not required to separate glacier entities with drainage divides derived from a watershed analysis of a DEM. Glacier-specific calculations can only be performed when the glacier mask is divided into entities by drainage divides. In a first step, a single glacier inventory is already highly useful as it allows selecting glaciers by size, using their mean elevation as a proxy for the balanced-budget equilibrium line altitude ELA_0_ (Braithwaite and Raper 2010) and henceforth precipitation amounts (e.g., Sakai et al. 2015), or calculating their ice-thickness distribution (Huss and Farinotti 2012; Linsbauer et al. 2012; Frey et al. 2014) or length (Kienholz et al. 2014; Machguth and Huss 2014). When glacier-specific outlines are combined with elevation changes derived from DEM differencing and an appropriate assumption for density (e.g., Huss 2013), it is possible to not only determine the sea level equivalent contribution of an entire region (e.g., Rignot et al. 2003; Schiefer et al. 2007; Berthier et al. 2010), but also the mean changes for each individual glacier. This so-called geodetic balance can be compared to cumulative field data and is used for the calibration of the in situ measurements. It further provides additional insights to the representativeness of the glaciers selected for field measurements for the entire region (Paul and Haeberli 2008; Le Bris and Paul 2015). On a larger scale, such a separation of contiguous ice masses by hydrologic drainage divides is also required to separate the glaciers and ice caps surrounding the Greenland Ice Sheet from the ice sheet and thus determine their mass change separately (Rastner et al. 2012).

When glacier outlines are available from at least two points in time, all kinds of associated changes can be derived. Whereas area changes themselves might only be indicative of mass changes, there are several further parameters that can be used to estimate corresponding mass changes. One is the change in minimum elevation that is—in a first-order approximation—two times higher than the change in mean elevation and thus the balanced-budget ELA (Haeberli and Hoelzle 1995). Combined with a mass balance gradient and the area-elevation distribution (hypsometry) of a glacier, the resulting mass changes can be determined, albeit only for a decadal time scale where measured changes are significant.

Finally, estimates of the mass balance may be obtained by determining the accumulation area ratio through remote sensing (Rabatel et al. 2008; Barandun et al. 2015). However, because of the required manual delineation of the snow line, this is not done frequently.

#### Derivation of Glacier Outlines and Trends from Satellite Data

**Fig. 3:**
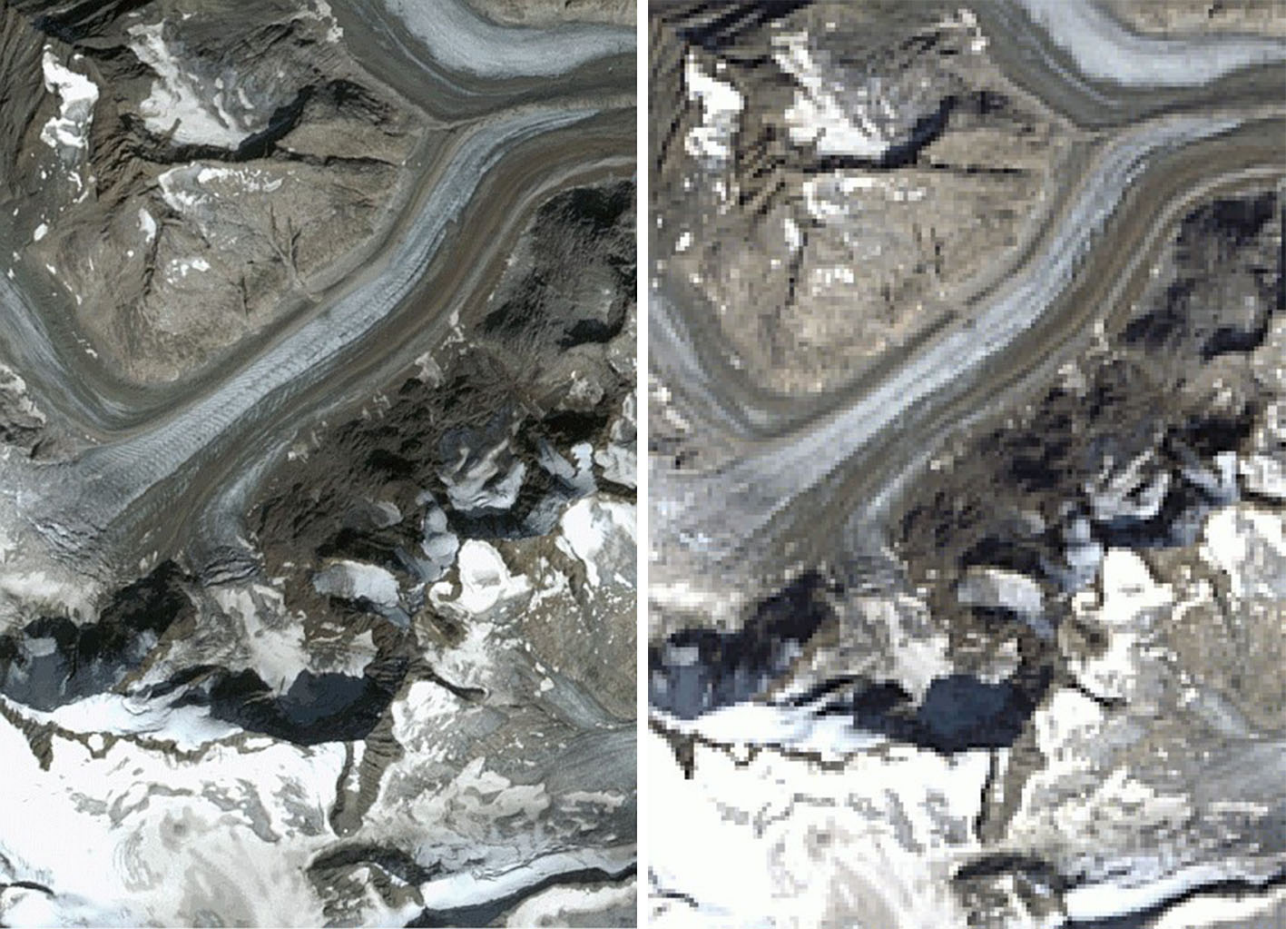
Comparison of a Sentinel 2 image (*left*) with 10 m resolution (acquired on 29 August 2015) to a Landsat 8 image (*right*) with 30 m resolution (31 August 2015) for the southern part of Unteraarglacier in the Swiss Alps. With Sentinel 2 fine spatial details of glacier flow become visible (ogives) and debris-covered regions can be identified and delineated more precisely. Sentinel data: Copernicus 2015, Landsat data: USGS

Automated glacier mapping with optical satellite images is straightforward when cloud-free scenes from the end of the ablation period or dry season (with minimal snow cover) are available. Snow and ice have a very low reflectance in the shortwave infrared (SWIR), whereas reflectance is much higher in the visible and near-infrared (VNIR). Hence, dividing reflectances in a VNIR band by those in a SWIR band gives high values over glaciers and low ones over all other terrain where lower reflectance values are divided by higher ones when using a red/SWIR band ratio. With a simple threshold this ratio image can be segmented into a binary glacier map and transformed into glacier outlines using raster-vector conversion. When abundant small snow patches are present, a 3-by-3 (or 5-by-5) median filter applied to the binary glacier map is useful for eliminating them and reducing noise (Paul et al. 2002; Rastner et al. 2012). The manually selected threshold value is most sensitive in regions of shadow and should be optimized for this region in a way that workload for corrections is minimized. Manual editing is required for debris-covered glacier parts as these have the same spectral reflectance as the surrounding terrain and can thus not be mapped based on spectral reflectance alone (Paul et al. 2004a, b). Apart from debris cover, clouds and seasonal snow are the main bottleneck for a fast and frequent production of glacier outlines (Paul et al. 2011). All three obstacles will likely improve when Sentinel 2A and 2B provide 10 m resolution images at least every 5 days (more often toward higher latitudes). Whereas the high repetition rate will help in acquiring cloud-free images from the real end of the ablation period, the high spatial resolution will help in identifying snow patches and delineating glaciers with debris more precisely (Fig. 3).

Thanks to the opening of the Landsat archive by USGS (Wulder et al. 2012), numerous studies have mapped glacier extents over large regions with the method described above (Bolch et al. 2010; Rastner et al. 2012; Frey et al. 2012; Guo et al. 2015) and contributed to the global completeness of the RGI (Pfeffer et al. 2014). The body of literature on regional glacier area changes revealed a globally consistent picture of shrinking glaciers over the past 50 years with rates mostly between 0.05 and 1% area loss per year (Vaughan et al. 2013). In some regions (Alps, Low Latitudes), they are even higher (up to 2%) and the majority of investigated regions showed a recent increase of area loss rates.

### Elevation and Volume Changes

Surface elevation changes can be used together with the glacier outline to determine volume changes for a given time period. To convert the measured volume changes to changes in mass, a density must be assigned. It is important to note that surface elevation changes and thereby volume changes do not necessarily mean a loss or gain in mass since they do not consider firn compaction or internal refreezing. Thus, by themselves, these parameters cannot be directly transferred to a sea-level budget.

There are three main methods used for determining surface elevations and thereby surface elevation changes of glaciers from satellite measurements. These are: repeat altimetry (Csathó et al. 2014), DEM differencing (Wang and Kääb 2015), and a combination of these two (Nuth et al. 2010; Helm et al. 2014). As shown in Table 1, each of the methods has advantages and disadvantages with regards to temporal and spatial coverage. The combination of altimetry and DEMs allows for the advantages of both methods to be exploited (e.g., Kääb et al. 2012). Combining multiple data sources where available, be it satellite, airborne or ground-based, gives the best possible assessment of change (Schenk and Csathó 2012; Csathó et al. 2014).

Both laser and radar have been used in past and current altimetry missions. Laser has a shorter wavelength and thus has the advantage of having higher resolution, but clouds reflect energy at this frequency so that the surface below is not measured when clouds are present. Radar is not affected by clouds, so the surface can always be measured. However, at the typical frequencies used, C-band to Ku-band, the energy penetrates into snow and ice, meaning that the distance measured may correspond to a region below the true surface. The amount of penetration and subsequent contribution of volume scattering to the signal depends both on the electromagnetic frequency of the signal and on the characteristics of the target material, in particular on liquid water content. It is greatest over the dry snow zones of the ice sheets, but most variable and therefore most difficult to correct for over glaciers and ice caps (e.g., Rignot et al. 2001; Müller et al. 2011; Gray et al. 2015).

ICESat was the first laser altimetry mission, operational from 2003 to 2009. Currently, an ICESat2 mission (Abdalati et al. 2010) is planned for launch in late 2017. There are currently 6 radar altimetry satellites in operation (http://www.altimetry.info/missions/). Of these, Cryosat-2 was the first to be dedicated to measuring changes in the cryosphere and is novel in that it has both SAR and Interferometric capabilities to improve both along and across track resolution. Altika is novel in that it operates at Ka-band frequency, presenting less of a surface penetration problem. To derive elevation changes, the commonly used cross-over method and the repeat-track method are suitable for both laser and radar missions (e.g., Moholdt et al. 2010).

Comprehensive coverage of individual glaciers is difficult with the relatively sparse coverage of altimetry missions such ICESat, although with swath processing of Cryosat-2 data this is no longer an issue (Gray et al. 2013). However, using a DEM for the surface elevation at one point in time and an altimetry dataset for the elevation at a second point in time allows all altimetry data points to be used as opposed to just those with repeat or cross-tracks. Use of a radar-based DEM, such as the widely used SRTM DEM, requires consideration of the penetration of the radar signal and the impacts this may have on the deduced surface elevation changes (Kääb et al. 2012).

Prior to differencing two DEMs, it is critical that they are co-registered to ensure the elevation change is calculated for corresponding ground points. Despite good co-registration, biases may remain due to acquisition strategy (Berthier et al. 2007) or DEM creation (Nuth and Kääb 2011), which will propagate to the final difference DEM if they cannot be corrected for. The final difference DEM will only be as good as the poorest input DEM. The most obvious manifestation of this is that no-data pixels, or voids, in one of the DEMs will produce voids in the resultant elevation change grid. Additional errors include noise and biases and can be evaluated by making statistical analyses of the surrounding stable terrain (e.g., Nuth and Kääb 2011).

The time stamps on the DEMs being differenced is a key part of any interpretation of the derived elevation change. Depending on the time difference, the calculated elevation changes can reveal short-term dynamic behavior, seasonal changes, annual or multi-decadal changes. Since the whole glacier system can be assessed, for land-terminating glaciers, the dynamic component of ice submergence and emergence cancels out. Thus, the DEM differencing method provides a possibility to assess climatic-induced elevation changes where sufficiently long time series are available. A time period of at least five years is recommended in order to avoid seasonal and small scale fluctuations, although the ideal period will also depend on the accuracy of the DEMs and the magnitude of the change being measured.

The recent sub-meter resolution optical satellites such as Quickbird, WorldView and Pléiades provide high-resolution spaceborne DEMs that push the spatial and temporal limits of what can be achieved with spaceborne DEM differencing (Kronenberg et al. 2016; Melkonian et al. 2016; Berthier et al. 2014).

### Glacier Mass Change from Spaceborne Gravity Observations

Glacier mass changes can be estimated at the global scale since the launch of the Gravity Recovery And Climate Experiment (GRACE) twin satellites in summer 2002. This mission is devoted to the observation of relative Earth gravity changes. On a monthly timescale, changes in gravity observed from space are mainly caused by changes in the mass of surface water (Wahr and Molenaar 1998). Due to the mission design and satellite orbit (polar orbit at an altitude of 500 km), Earth gravity changes are delivered as monthly spherical harmonic coefficients at the Earth surface with a spatial resolution around 300 km by the three data centers responsible for the GRACE mission (Geoforschungszentrum Postdam, GFZ; Center for Space Research at University of Texas, CSR; Jet Propulsion Laboratory, JPL; Wahr and Molenaar 1998; Jacob et al. 2012; Sakumura et al. 2014). The most recent gravity field from GRACE is the Level 2 Release 05 (RL05), which includes de-aliasing processing. Sakumura et al. (2014) have shown that using the arithmetic mean of all gravity field solutions is the most effective in reducing the noise.

All the methods to estimate glacier mass changes from spaceborne gravimetry observations convert the spherical harmonic coefficients (Stokes coefficients) into surface water thickness equivalent and finally into mass changes using the formulation presented in Wahr and Molenaar (1998). The main challenge remains to attribute the observed mass changes to glaciers and other mass change sources. Two recent methods exist to do this: the first is the mass concentration blocks approach (Jacob et al. 2012; Schrama et al. 2014). This attempts to determine the mass change of glacierized regions by fitting mass values (i.e., a set of Stokes coefficients) obtained from the gravity field measured by GRACE into small regions (called mascons) where the mass is assumed to be uniformly distributed. This approach can be applied to Level 2 GRACE data or also to raw Level 1 measurements. The second method is the forward modeling approach (Chen et al. 2013; Yi et al. 2015) that is based on an iterative and global forward modeling of the Stokes coefficients, in order to separate the terrestrial from the ocean signal. Known locations of terrestrial mass sources can be used to constrain mass changes. In this approach, the total mass on the Earth surface is conserved.

Note that usually during the attribution of mass changes to regions with glaciers and ice caps, the Stokes coefficients of degree 2 and order 0, as well as the degree 1 are, respectively, replaced with those from satellite laser ranging and those estimated based on Swenson et al. (2008). In addition, a correction for glacial isostatic adjustment (GIA) and a Gaussian filter (usually 300 km large or more) is applied.

Despite recent method improvements, uncertainties are still important in glacier mass changes estimated from GRACE measurements, especially due to the heterogeneity and the small size of glaciers compared to the spaceborne gravimetry spatial resolution, which is determined by satellite altitude and distance between the two twin satellites (Wahr and Molenaar 1998). For example, there is significant leakage of the glacier mass signal into the oceans. Excluding coastal regions, or using the forward modeling approach is useful to solve this issue. However, these solutions are not completely satisfying because they are based on questionable assumptions, such as a uniform distribution of mass change in the ocean. Another example of uncertainty is the difficulty to distinguish between mass change originating from land water storage changes and glacier mass changes. Using known spatial patterns (glacier or river basins, mascons, etc.) or hydrological modeling can be a basis for removing the land water contribution from the signal. However, the spatial separation is not clear everywhere, especially in regions where the land water storage signal or annual cycle is large (e.g., Himalaya regions, Andes region) and where hydrological models show large discrepancies. Finally, the necessity to remove the GIA signal from GRACE-based mass changes causes uncertainty (Schrama 2016). The GIA signal does not include more recent effects, e.g., the rebound from the Little Ice Age, which is essential in some glacierized areas like Patagonia or Alaska (Larsen et al. 2005; Jacob et al. 2012).

**Fig. 4:**
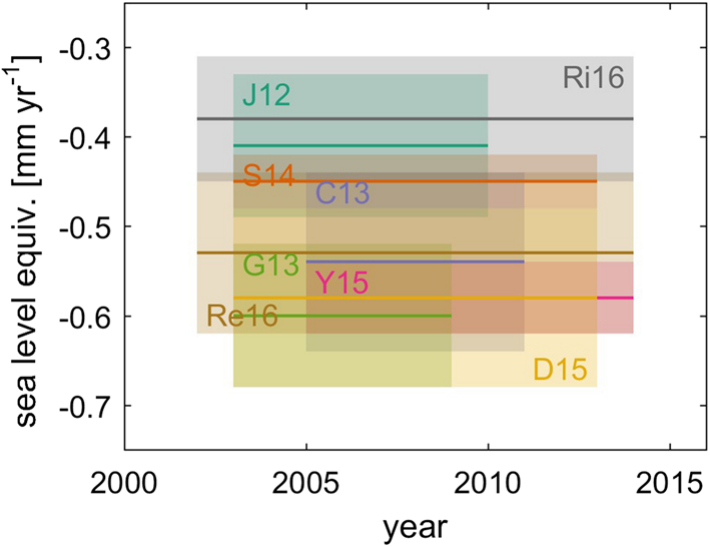
Glacier mass change estimates based (partly) on GRACE data. Boxes indicate period covered and upper and lower confidence level of estimate. J12 is Jacob et al. (2012), G13 is Gardner et al. (2013), C13 is Chen et al. (2013), S14 is Schrama et al. (2014), D15 is Dieng et al. (2015), Y15 is Yi et al. (2015), Re16 is Reager et al. (2016) and Ri16 is Rietbroek et al. (2016)

**Fig. 5:**
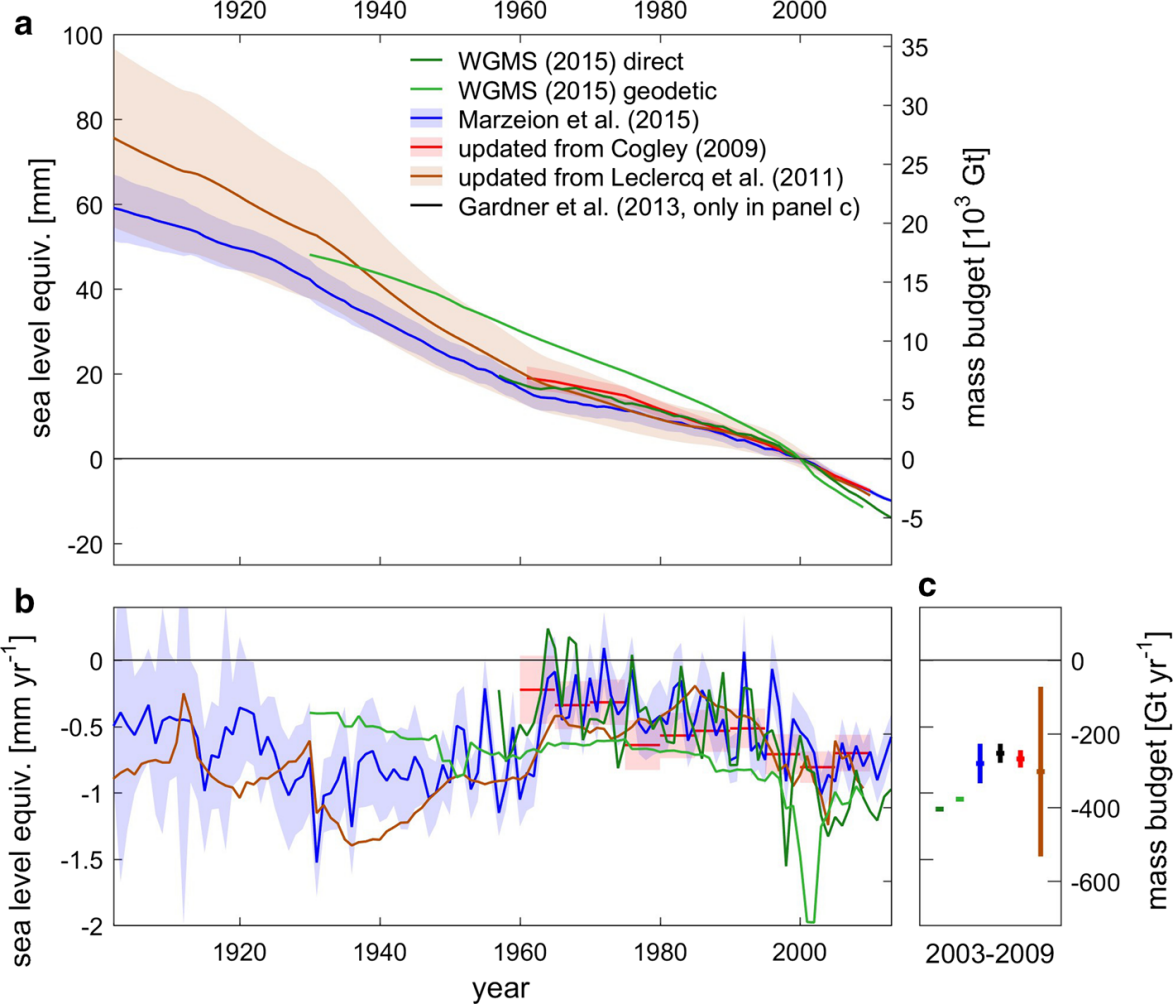
Globally integrated glacier mass change: **a** accumulated in time, relative to the year 2000; **b** mass change rates; **c** mean mass change rates during 2003 to 2009. *Shading* and *error bars* indicate the 90% confidence interval. Note that all values exclude glaciers in the Antarctic periphery. For the time series labeled WGMS, the global mean of direct and geodetic specific mass balance measurements from Zemp et al. (2015, data from WGMS 2015) were multiplied with the global glacier area of Pfeffer et al. (2014). Figure extended from Marzeion et al. (2015)

The most recent global estimates of glacier mass changes include Jacob et al. (2012) with 0.41 ± 0.08 mm SLE year^−1^ (2003–2010 time period) and Schrama et al. (2014) with 0.45 ± 0.03 mm SLE year^−1^ (2003–2013) using the mascon-based approach, Chen et al. (2013) with 0.54 ± 0.10 mm SLE year^−1^ (2005–2011) and Yi et al. (2015) with 0.58 ± 0.04 mm SLE year^−1^ (2005–2014) using the forward modeling-based method, and the four following studies which combined GRACE-based estimates with other datasets (e.g., altimetry, literature assessment, sea-level budget approach): Gardner et al. (2013) with 0.60 ± 0.08 mm SLE year^−1^ (2003–2009), Dieng et al. (2015) with 0.58 ± 0.1 mm SLE year^−1^ (2003–2013), Reager et al. (2016), an update of Gardner et al. (2013), with 0.53 ± 0.09 mm SLE year^−1^ (2002–2014) and Rietbroek et al. (2016) with 0.38 ± 0.07 mm SLE year^−1^ (2002–2014). See Fig. 4 for a comparison of these estimates. Because of the high temporal variability (see Fig. 5) and its influence on the estimated trends (Yi et al. 2015), all these results are difficult to compare with one another. However, most values are consistent and show an average rate of glacier mass losses of 0.51 ± 0.07 mm SLE year^−1^ for the last decade (2002/2005–2013/2015), which represents 184 Gt of fresh water added into the ocean every year. GRACE-based glacier mass losses are smaller than those derived from in situ measurements or modeling estimates. Note that peripheral glaciers in Antarctica and Greenland (excluded from the studies above) were estimated to additionally have contributed 0.02 ± 0.03 mm SLE year^−1^ and 0.1 ± 0.02 mm SLE year^−1^, respectively, during 2003–2009 (Gardner et al. 2013).

## Modeling Glaciers Based on Climate Observations

While comprehensive monitoring of glaciers on the global scale only became available with modern remote sensing methods, the state of the global atmosphere has been observed by a dense (at least compared to glacier observations) network of weather stations for many more decades. Based on objective analysis and homogenization of individual records, Harris et al. (2014) present a monthly, globally complete gridded dataset of temperature and precipitation starting with the beginning of the twentieth century. Datasets like this (similarly, global reanalysis data) allow another observation-based estimate of past glacier change: instead of relying on observations of glaciers, observations of the state of the atmosphere may be used as boundary conditions for glaciological modeling. However, most published global glacier models rely on this reconstructive mode of operation only for purposes of model validation and calibration, neglecting any past glacier geometry change (e.g., Radić et al. 2014; Huss and Hock 2015).

However, on time scales of decades and more, glacier geometry change presents several important feedbacks to glacier mass balance (e.g., Paul 2010). On the one hand, the stabilizing, negative feedback of glacier advance and thus progression to a warmer environment during periods of mass gain, and the retreat to higher, cooler altitudes during periods of mass loss, are important. On the other hand, particularly the largest glaciers with flat and thick tongues often only have weakly inclined or even retrograde beds. This leads to down-wasting rather than dynamically active retreat to higher elevations and may prevent a recovery of the glacier tongue once the surface lowering has started. Because of the delayed response of glaciers to climate forcing, these feedbacks are not simple to capture in glacier models in reconstructive mode: to estimate the past glacier geometry, the past mass balance has to be known, which itself is a function of the past geometry. Marzeion et al. (2012, updated in Marzeion et al. 2015), developed an iterative procedure to identify the state of a glacier in the year of model initialization (e.g., 1901) that will result in the observed state of the glacier in the year of observation (e.g., 2005) after a forward model run, using atmospheric conditions from Harris et al. (2014) as boundary condition. They then used the RGI to reconstruct past changes of each of the world’s glaciers, using direct glacier observations for model calibration and cross-validation.

While this approach benefits from the relative abundance of atmospheric observations, it suffers from the additional uncertainty introduced by the glacier model. Moreover, the validation with direct glacier observations is of limited value for uncertainty estimates in the earlier period of the reconstruction, as most direct glacier observations were taken at times and in places where the observations of the state of the atmosphere can be assumed to be of above-average quality and quantity compared to other remote and mountainous regions.

## Synthesis and Discussion

Direct and geodetic measurements of glacier mass change, calibrated reconstructions of mass change based on glacier length change records and modeled reconstructions forced by climate observations are all available for (at least) several decades. Over the past years, progress in data availability and methodological advances has led to greater consistency in the results of the respective methods (Marzeion et al. 2015). In Fig. 5, we compare the most recent results for the twentieth century. In Table 2, we additionally list estimates based on remote sensing for a more recent period.

**Table 2:** Comparison of mass change estimates during common periods, in mm SLE year^−1^ and the 90% confidence interval, where given in the source

Source	2003–2009	1961–2010	1901–2010
WGMS (2015) direct	−1.12	−0.57	–
WGMS (2015) geodetic	−1.05	−0.85	–
Marzeion et al. (2015)	−0.78 ± 0.15	−0.49 ± 0.05	−0.62 ± 0.05
Updated from Cogley (2009)	−0.75 ± 0.07	−0.54 ± 0.05	–
Updated from Leclercq et al. (2011)	−0.84 ± 0.64	−0.58 ± 0.15	−0.78 ± 0.19
Gardner et al. (2013)	−0.70 ± 0.07	–	–
Average of GRACE-based studies, see Sect. 3.3 for sources	−0.61 ± 0.07^a^	–	–

^a^Averaged over different time periods (2002/2005–2013/2015) and adding the estimate for Greenland peripheral glaciers from Gardner et al. (2013)

Over multi-decadal time periods, the greatest deviation is between the estimates based on the arithmetic means of direct and geodetic observations (WGMS 2015) on the one hand, and the estimates based on glacier length change observations (Leclercq et al. 2011), glacier modeling (Marzeion et al. 2015) and based on spatially weighted observations (Cogley 2009) on the other hand. Since the data used by Cogley (2009) and WGMS (2015) are to a large degree identical, this discrepancy can probably be explained by non-representative sampling of the glacier observations. Specifically, glacier observations appear to be made on glaciers that tend to have more negative mass balances than the global, or even regional, mean (Gardner et al. 2013, also found indications of this).

During the more recent years when estimates from remote sensing are available, it becomes apparent that they consistently indicate weaker glacier mass losses, even when taking into account the different regions considered (i.e., results from GRACE generally exclude glaciers in the Greenland and Antarctic peripheries). While the error margins allow a reconciliation of most of these estimates, it needs to be better understood where the systematic differences originate.

It is important to consider that not all meltwater from shrinking or vanishing glaciers directly contributes to sea-level rise (Haeberli and Linsbauer 2013; Loriaux and Casassa 2013). Some glacier parts are below sea level and, because of the ice/water density difference, even cause a slight lowering of the sea level when replaced by water. Meltwater from glaciers on land may be held back in lakes, which form in over-deepened parts of glacier beds when becoming exposed. On its way to the ocean, glacier meltwater on land can be lost by natural processes or be used through human activity (Bury et al. 2013; Carey et al. 2014a, b). Some rivers fed by glacier meltwater drain endorheically and do not reach the ocean at all (Kääb et al. 2015; Neckel et al. 2014). This effect may in fact be a partial explanation for the difference between GRACE-based versus other estimates: GRACE integrates large regions and thus includes in its glacier mass loss estimates the increase of water stored in lakes or in the ground. Coastal effects such as shoreline migration, changes in ocean area and isostatic adjustments to land and ocean surfaces must also be considered (Huss and Hock 2015).

Scientific research into the questions mentioned above is at its very beginning and relates to complex systems. In recent years, the development of flux/stress/slope-related approaches to calculate detailed ice thicknesses and glacier-bed topographies (Haeberli 2016) enabled coherent and well-constrained calculations of glacier volumes and first estimates of ice below sea level and glacier overdeepenings. Based on detailed worldwide ice-thickness modeling, Huss and Farinotti (2012) provided a value of 0.43 ± 0.06 m sea-level equivalent for the total ice volume. Haeberli and Linsbauer (2013) used information from modeled glacier-bed topographies for still existing mountain glaciers (Linsbauer et al. 2012 for the Swiss Alps; cf. also Linsbauer et al. 2015 for the Himalaya–Karakoram region) to infer that ice below sea level and in overdeepenings together accounts for a few (probably 1–6) centimeters SLE, with millimeters rather than centimeters in overdeepenings and centimeters rather than millimeters below sea level. This is confirmed by Huss and Hock (2015) who re-calculated the total glacier volume to be 37.4 cm SLE and that 11–14% of the ice volume to be lost in the twenty-first century is already below sea level (glacier-bed overdeepenings at higher elevations not included). Not even order-of-magnitude assessments are available for the amount of meltwater being diverted over land. The evolution in time is likely different for the various components of glacier volume. The amount of ice below sea level is likely to decrease in the twenty-first century due to the retreat of glaciers out of the ocean (Haeberli and Linsbauer 2013; Huss and Hock 2015). However, the amount of meltwater stored in overdeepenings may increase over time even into the twenty-second century.

There is another systematic error likely leading to an underestimation of past glacier mass loss, which is based on the temporal limitation of the glacier inventories used for upscaling measurements or initializing models. These inventories do not necessarily contain information on glaciers that have already disappeared but produced meltwater during their disappearance. As these are necessarily small glaciers, their contribution to the total mass loss is likely also small. But whereas estimates exist on the number and size of small glaciers missing in state-of-the-art inventories (Pfeffer et al. 2014 estimate an upper bound of the missing glaciers at 1.1–1.4% of global glacier area), we are not aware of any estimate of their potential past mass loss. However, Bahr and Radić (2012) find that their contribution to the total glacier mass is probably non-negligible, and it is particularly for these small glaciers, that the option of “saving the glaciers” hardly exists anymore (see Sect. 2.1).

In conclusion, an integrative observational strategy combining in situ measurements, satellite observations and numerical modeling is desirable to provide a comprehensive view of past and ongoing glacier change. To bridge the gap between detailed local investigations and global coverage, a tiered strategy has already been developed (Haeberli et al. 2000; WGMS 2015), including:

- process understanding and model development/calibration/assimilation
  - extensive measurements of energy/mass balance, flow, etc.
- regional indicators
  - mass change (index stakes + photogrammetry, LIDAR)
- regional representativeness
  - cumulative length change, DEM differencing
- global coverage
  - inventories (remote sensing/geoinformatics)

To this end, field measurements of mass balance will remain essential in the future to (a) separate surface effects from effects of glacier flow (submerging/emerging flow, etc.) and, hence, (b) understanding of, and numerical model development for, mass and energy balance processes. The full implementation of the above strategy will likely result in more reliable, spatially and temporally well-resolved data on glacier mass change. Ideally, it could lead to similar agreement between the different methods on regional scales as can now be found on the global scale, which would be an important step to better understanding glacier mass changes at a regional scale.

## Footnotes

1 Note that glacier mass loss does not equal ocean mass gain, as not all melt water may end up in the ocean (cf. Sect. 5).
